# Bone Marrow Mesenchymal Stem Cells Reversed Ovarian Aging-related m6A RNA Methylation Modification Profile in Aged Granulosa Cells

**DOI:** 10.1007/s12015-022-10485-y

**Published:** 2023-01-07

**Authors:** Chuan Tian, Yuanyuan An, Jing Zhao, Xiangqing Zhu, Wei Wei, Guangping Ruan, Ye Li, Xinghua Pan

**Affiliations:** 1 The Basic Medical Laboratory of the 920Th Hospital of Joint Logistics Support Force of PLA, The Transfer Medicine Key Laboratory of Cell Therapy Technology of Yunan Province, The Integrated Engineering Laboratory of Cell Biological Medicine of State and Regions, Kunming, 650032 Yunnan Province China; 2 The Affiliated Stomatology of Kunming Medical University, Kunming, 650106 Yunnan Province China

**Keywords:** BMMSCs, Ovarian ageing, m6A, Lysine demethylase 8 (KDM8)

## Abstract

**Background:**

Ovarian ageing causes endocrine disturbances and the degeneration of systemic tissue and organ functions to seriously affect women's physical and mental health, and effective treatment methods are urgently needed. Based on our previous studies using juvenile rhesus monkey bone marrow mesenchymal stem cells (BMMSCs) to treat ovarian ageing in rhesus monkey, we found that BMMSCs improved ovarian structure and function. This study continues to explore the mechanism by which BMMSCs reversed granulosa cell (GC) ageing.

**Methods:**

A GC ageing model and coculture system of BMMSCs were established, changes in the level of the N6-methyladenosine (m6A) methylation modification were detected, m6A-modified RNA immunoprecipitation sequencing (MeRIP-seq) were performed, correlations between m6A peaks and mRNA expression were determined, and the expression of hub genes was identified using Q-PCR, immunofluorescence staining, and western blot.

**Results:**

Our results showed that H_2_O_2_ successfully induced GC ageing and that BMMSCs reversed measures of GC ageing. BMMSCs increased the expression of the FTO protein and reduced the overall level of m6A. We identified 797 m6A peaks (348 hypomethylated and 449 hypermethylated peaks) and 817 differentially expressed genes (DEGs) (412 upregulated and 405 downregulated) after aged GCs were cocultured with BMMSCs, which significantly associated with ovarian function and epigenetic modification. The epigenetic repressive mark and important cell cycle regulator lysine demethylase 8 (KDM8) was downregulated at both the mRNA and protein levels, histone H3 was upregulated in aged GCs after BMMSC coculture, and KDM8 was upregulated after FTO was inhibited through FB23.

**Conclusions:**

Our study revealed an essential role for m6A in BMMSCs in reversing GC ageing, and FTO regulated KDM8 mediates histone H3 changes may as a novel regulatory mechanism in BMMSCs to reverse GC ageing.

**Graphical Abstract:**

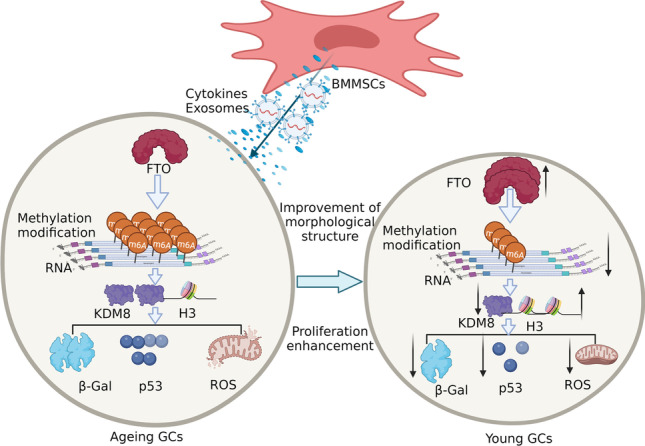

**Supplementary Information:**

The online version contains supplementary material available at 10.1007/s12015-022-10485-y.

## Introduction

The world's population is ageing rapidly, and the number of elderly individuals has increased significantly, which has placed great pressure on society and become one of the major economic challenges facing contemporary society [1–3]. The ovary is a dynamic reproductive endocrine organ that enacts female reproductive function through ovulation and the secretion of sex hormones and affects tissues and organs throughout the body [4, 5], it is also one of the most sensitive organs to ageing. A variety of stimuli may lead to ovarian ageing, which makes women infertile and is potentially accompanied by related growth and developmental diseases that are serious threats to women's health [6–8]. At the molecular level, ovarian ageing is gradual and involves multifactor interactions and complex biological processes; it is caused by decreases in follicle quantity and quality and is related to autoimmunity, genetic susceptibility, mitochondria, and telomerase [9–11]. However, the mechanisms of transcription, regulation and modification of reproductive helper cells in the occurrence and development of ovarian ageing are not clear, which hinders the progress of effective treatment of ovarian ageing.

The ageing ovary is mainly characterized by tissue structure atrophy, lack of self-renewal ability of reproductive helper cells, decreased cell numbers and functional degeneration. Bone marrow mesenchymal stem cells (BMMSCs) have several biological characteristics: multidirectional differentiation potential, strong self-renewal ability, and the ability to secrete multiple cytokines and repair tissue [12, 13]. Therefore, they may become a novel tool for reversing ovarian ageing. Many studies have shown that mesenchymal stem cells (MSCs) are safe and effective in the treatment of ovarian ageing, and they are a more effective cell type to improve ovarian structure and function [14]. Previous studies have confirmed that MSCs can regulate women's sex hormone secretion, promote follicular regeneration, and restore the activity and number of reproductive helper cells [15–18]. However, the transcriptional modification profile and key regulatory signaling pathways in MSCs used in the treatment of ovarian ageing are unclear, and systematic research with comparisons to normal controls is lacking.

N6-methyladenosine (m6A) is a common internal modification of mRNA and has many effects on the fate of mRNA [19]. Recent studies have found that m6A plays an important role in regulating gene expression, splicing, RNA editing, and RNA stability, controlling mRNA lifespan and degradation, and mediating circular RNA translation [20, 21]. In addition, previous studies have showed that m6A was significantly associated with ovarian ageing and ageing-related diseases [22–25]. However, researchers have not comprehensively investigated whether BMMSCs affect ovarian ageing by regulating the m6A modification of RNA, which attracted our attention.

Therefore, a human granulosa cell (hGC) ageing model was established and cocultured with BMMSCs in vitro to explore the mechanism by which MSCs participate in ovarian ageing. Then, m6A-modified RNA immunoprecipitation sequencing (MeRIP-seq) and bioinformatics analyses were performed to explore the overall effect of BMMSCs on m6A levels in RNA and the mRNA expression profiles, and the key factors and regulatory signalling pathways were identified using a variety of biotechnological approaches to provide a theoretical basis for the use of MSCs to treat ovarian ageing.

## Results

### H_2_O_2_-induced hGC Ageing, and BMMSCs Reversed hGC Ageing

**Fig. 1:**
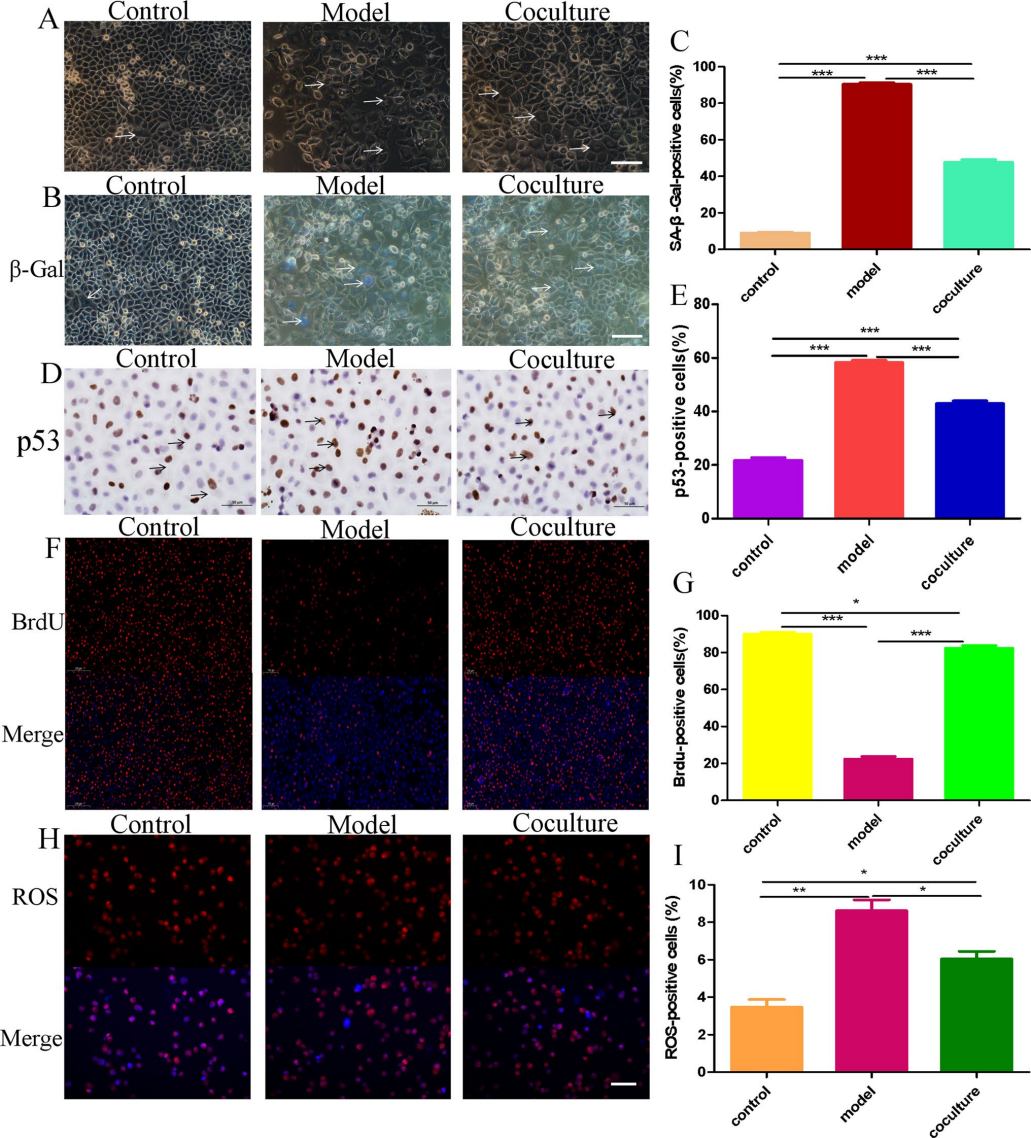
hGC ageing model and coculture with BMMSCs. **A** The morphology of GCs (200 ×). **B-C** β-Galactosidase staining detected the activity of β-galactosidase (200 ×). **D-E** Immunohistochemical staining detected the expression of P53(50 μm). **F-G** BrdU staining was performed to observe hGC proliferation and division (100 ×). **H-I** DHE staining detected the ROS level (200 ×). P < 0.05 indicates a statistically significant difference

hGCs are the most important cells in the ovary; therefore, an hGC ageing model was established in vitro to further explore the mechanism of BMMSCs in ovarian ageing. As a small-molecule oxidant, H_2_O_2_ easily causes cell ageing by inducing oxidative stress through the biofilm system, and this technique has been widely applied to induce cell ageing [26, 27]. In our study, hGCs were exposed to H_2_O_2_ for 24 h and cocultured with BMMSCs for 48 h. Firstly, under the fluorescence inverted microscope, the GCs in the model group were flat and wide, with a large number of intracellular vacuoles and a small number of cells compared with the control group. Meanwhile. the morphology of GCs was improved, the number increased and the intracellular vacuoles decreased after coculture with BMMSCs (Fig. 1A). Secondly, β-galactosidase staining (blue staining due to β-galactosidase activity) (Fig. 1B) showed that 7.33 ± 1.69% of the hGCs were stained blue in the control group, 93.33 ± 0.47% in the model group, and 43.66 ± 2.05% in the coculture group (Fig. 1C). Then, immunohistochemical staining was performed to detect the expression of the P53 protein (Fig. 1D), and the results showed that 21.04 ± 0.48% of cells expressed the P53 protein in the control group compared with 58.20 ± 1.21% in the model group and 42.90 ± 1.41% in the coculture group (Fig. 1E). Next, Proliferation and division reflect the activation of hGCs. BrdU staining (red) showed that hGCs were proliferating and dividing (Fig. 1F): 87.66 ± 1.24% of hGCs were stained red in the control group compared with 16.33 ± 1.24% in the model group and 80.66 ± 1.24% in the coculture group (Fig. 1G). Finally, reactive oxygen species (ROS) are an important ageing marker [28, 29], and DHE staining showed that the ROS level was 3.77 ± 0.12% in the control group, 8.63 ± 0.12% in the model group, and 5.63 ± 0.12% in the coculture group (Fig. 1H, I). Those results suggested that the hGCs were ageing after treatment with 273 mM H_2_O_2_ for 24 h, and BMMSCs reversed the changes in these ageing markers in aged hGCs.

## Changes in the Level of the m6A RNA Methylation Modification in Aged hGCs and After Treatment with BMMSCs

**Fig. 2:**
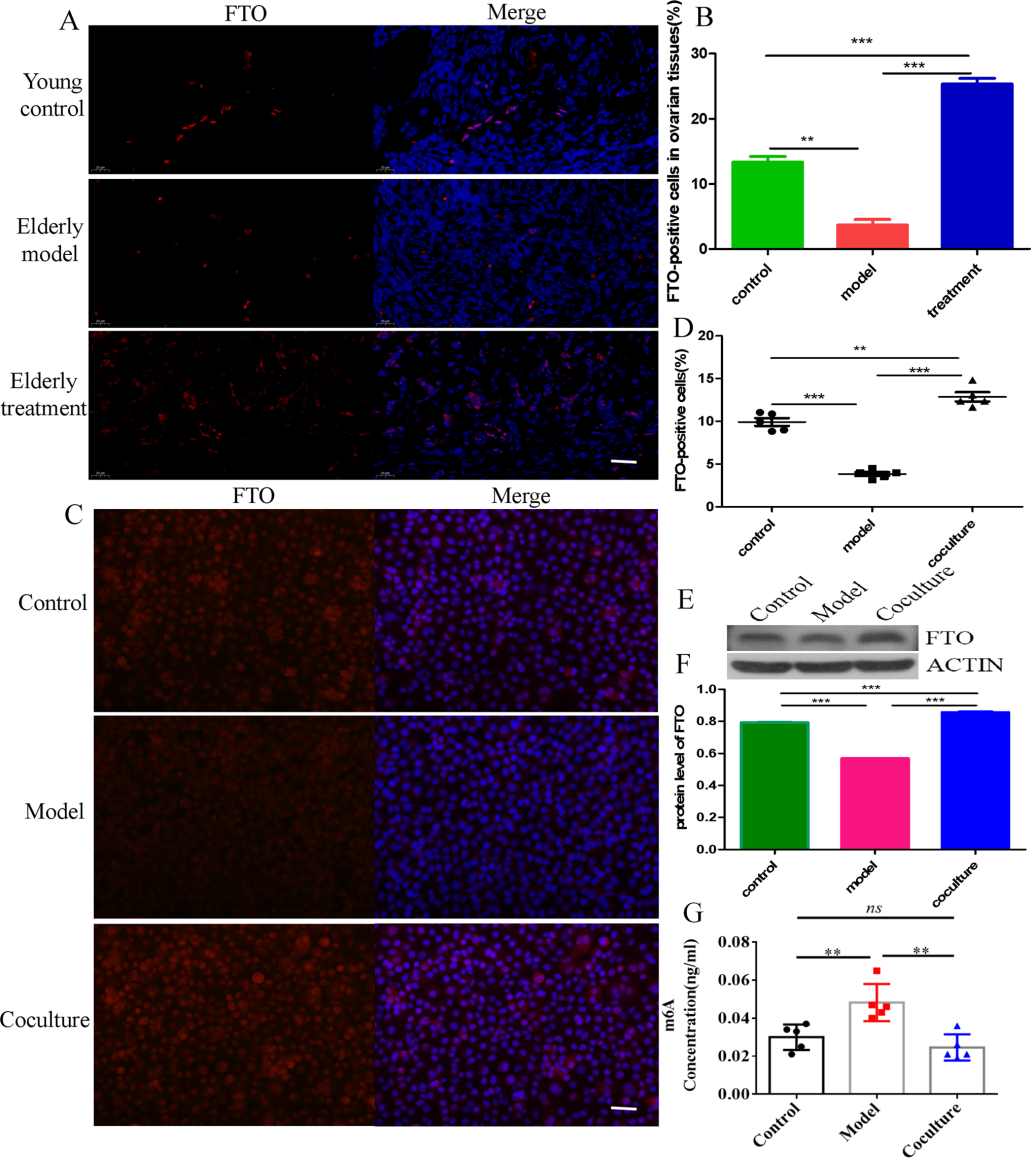
Changes in the level of the m6A methylation modification. **A-B** Immunofluorescence staining detected the expression of FTO in ovarian tissues(40 μm). **C-D** Immunofluorescence staining detected the expression of FTO in hGCs (400 ×). **E–F** Western blot showing the protein levels of FTO in hGCs.** G** Colorimetric detection of the overall m6A level

Studies have proven that the expression of the demethylase FTO is downregulated in ovarian tissue with increasing age to promote ovarian ageing [22, 30]. However, researchers do not adequately understand whether BMMSCs regulate FTO to alter methylation and subsequently reverse ageing. Interestingly, our results showed that the expression of the FTO protein was markedly upregulated in ovarian tissues of macaques after BMMSCs treatment compared with the model group (Fig. 2A, B). Furthermore, immunofluorescence staining indicated that FTO was significantly upregulated in aged hGCs after coculture with BMMSCs in vitro (Fig. 2 C, D), and this result also was confirmed by western blotting (Fig. 2E, F). In addition, the overall level of m6A was reduced in aged hGCs after coculture with BMMSCs (Fig. 2G), suggesting that the m6A methylation modification of RNA is closely related to hGC ageing, and BMMSCs play a key role in regulating FTO expression and the subsequent m6A methylation modification to reverse hGCs ageing.

## Overview of the m6A Methylation Landscape in Aged hGCs before and after Coculture with BMMSCs

**Fig. 3:**
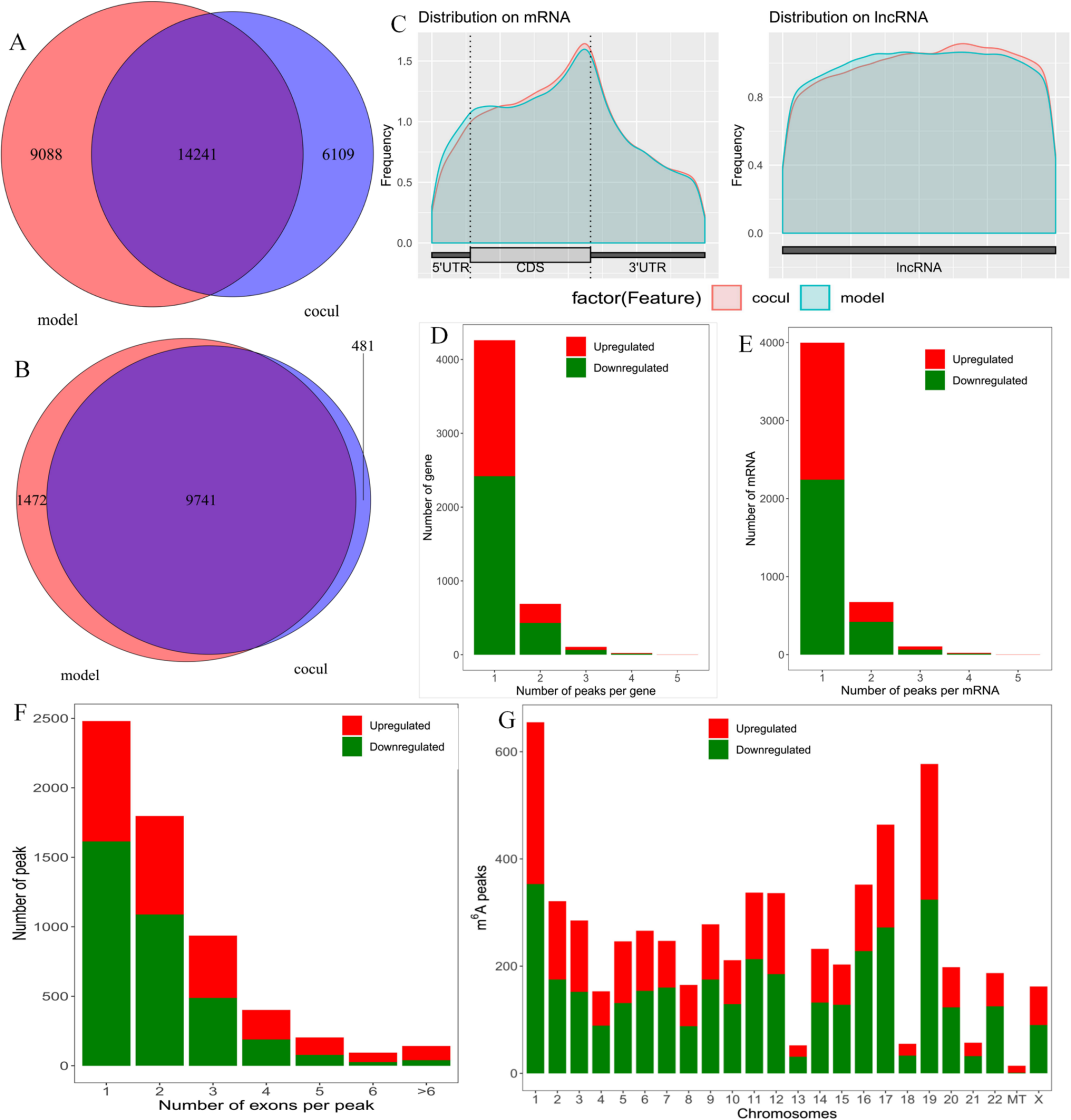
Overview of the m6A methylation landscape. **A** Venn diagram showing the m6A peaks. **B** Venn diagram showing the m6A-modified genes. **C** Distribution of m6A peaks in mRNA and lncRNA. **D** Distribution of altered m6A peaks per gene. **E** Distribution of altered m6A peaks per mRNA. **F** Distribution of altered m6A peaks per exon. **G** Distribution of altered m6A peaks in human chromosomes. Fold change ≥ 2 and P < 0.05

Notably, m6A is the most prevalent modification of mRNAs and lncRNAs and plays a key role in ageing and various ageing-related diseases [24, 31]. However, its specific regulatory mechanism in ovarian ageing remains unclear. In our study, after hGC ageing was induced and cells were cocultured with BMMSCs, MeRIP-seq was performed to explore the effect of BMMSCs on the m6A modification in ageing hGCs. Our results showed that 7,923 transcripts displayed a total of 14,417 sites that were modified by m6A in the model group, and 6,867 transcripts displayed a total of 11,715 sites that were modified by m6A in the coculture group. Among them, 14,241 individual m6A peaks in 9,741 m6A-modified genes were detected in the model and coculture groups (Fig. 3A, B). Notably, the coculture group had 6,109 new peaks and 9,088 missing peaks compared to the model group, revealing that the global m6A modification patterns were markedly different between the model and coculture groups (Fig. 3A). As shown in Supplementary Fig. 1A, B, the results showed different patterns of peaks with a relative increase in the start codon region (6.4 vs. 5.7% for aged hGCs and aged hGCs cocultured with BMMSCs, respectively) and in the 3’ untranslated region (3’UTR, 39.4 vs. 37.6%) and a relative decrease in the coding sequence (CDS, 30 vs. 32.3%) and at the stop codon (20.1 vs. 21%). Figure 3C shows that the distribution of m6A signals around mRNAs and lncRNAs was comparable in the model and coculture groups. In general, m6A peaks tended to occur in CDS regions and 3’UTRs, suggesting that m6A is likely to play a crucial role in regulating the expression and stability of mRNAs, consistent with previous MeRIP-seq results [32, 33]. The m6A peak distribution analysis suggested that most mRNAs and genes had m6A peaks, and there were mostly 1 to 3 m6A modifications in the exons (Figs. 3D, E, F). In addition, m6A peaks were found in all chromosomes, with the highest numbers being in chr1, chr17, and chr19 (Figs. 3G).

## Differences in m6A Peaks between Model and Coculture Groups

**Fig. 4:**
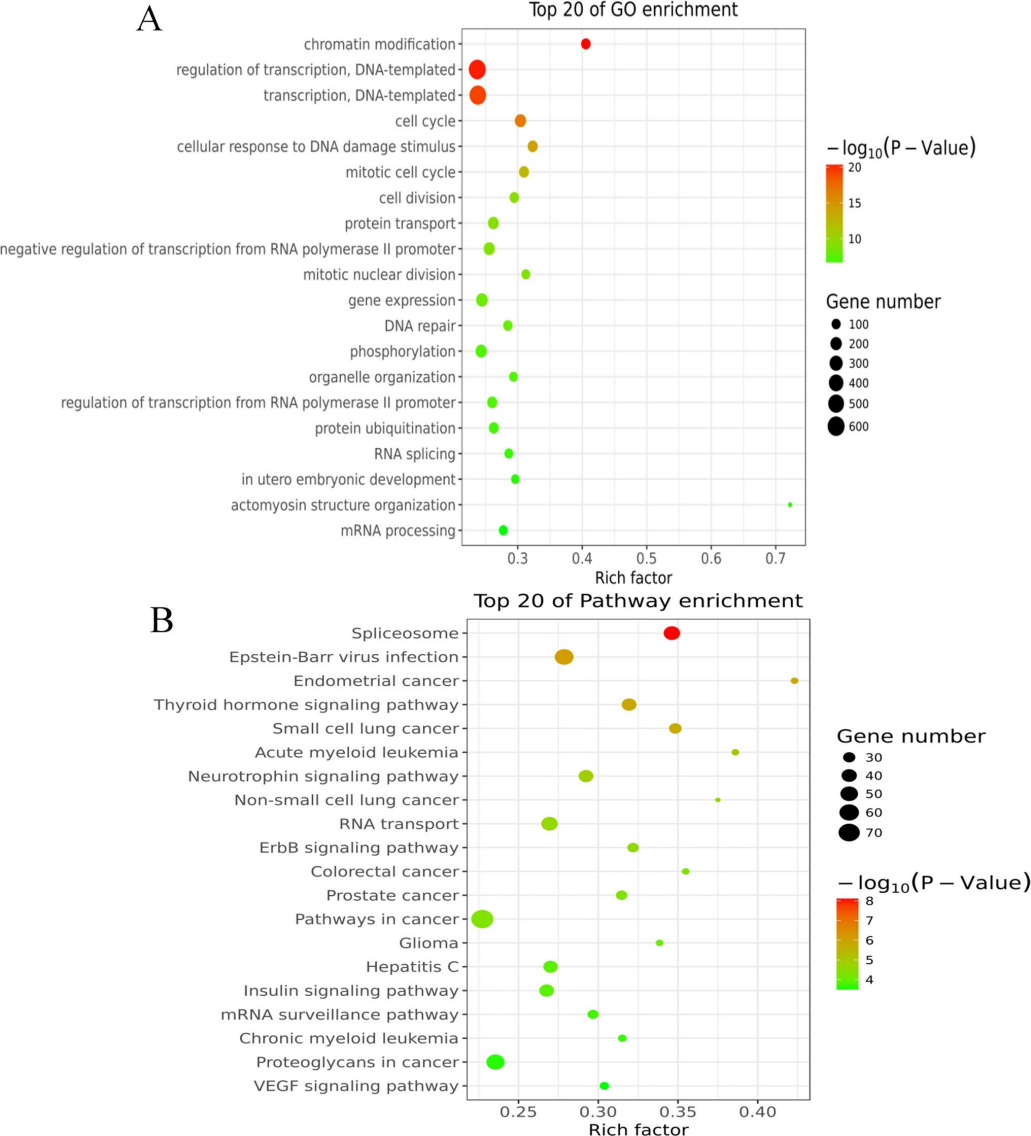
Biological significance analysis of differentially methylated mRNAs. **A** Bubble chart showing the top 20 enriched GO terms. **B** Bubble chart showing the top 20 enriched pathway terms

GO and KEGG pathway analyses of differentially methylated mRNAs were conducted to explore the biological significance of the m6A modification in BMMSCs interacting with aged hGCs. As shown in Supplementary Fig. 2A, compared to aged hGCs alone, aged hGCs that had been cocultured with BMMSCs presented 449 significantly upregulated m6A peaks and 348 downregulated m6A peaks (fold changes ≥ 2). Furthermore, the classic GGACU motif and the top 5 m6A motifs were observed in the model (Supplementary Fig. 2B) and coculture groups (Supplementary Fig. 2C). GO results showed that the altered m6A peaks were noticeably enriched in chromatin modification, regulation of transcription, DNA − templated, and cell cycle (Fig. 4A). Additionally, in KEGG pathway analyses, the spliceosome, Epstein-Barr virus infection, and thyroid hormone signalling pathway were markedly correlated with genes that showed m6A peaks in aged hGCs (Fig. 4B). Those results imply that BMMSCs reversed hGC ageing, which was significantly related to regulating the m6A methylation modification of mRNAs.

## Changes in mRNAs in Aged hGCs before and after Coculture with BMMSCs

**Fig. 5:**
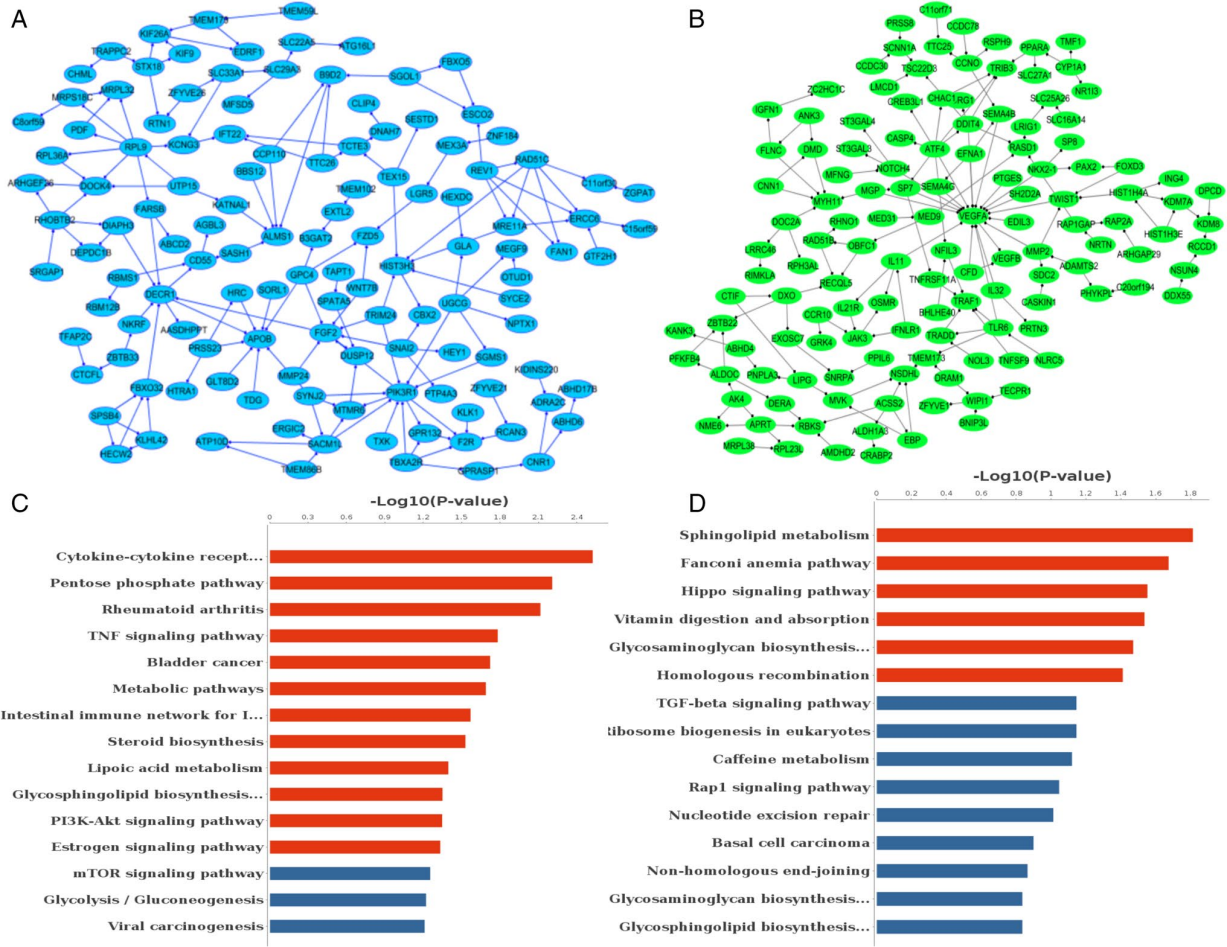
Analysis of the changes in transcriptome expression profiles between the model and coculture groups. **A** PPI networks of downregulated genes after coculture with BMMSCs. **B** PPI networks of upregulated genes after coculture with BMMSCs. **C** Top 20 pathways enriched in downregulated genes after coculture with BMMSCs. **D** Top 20 pathways enriched in upregulated genes after coculture with BMMSCs

First, we assessed the transcriptome profiles of altered genes in three pairs of aged hGCs and aged hGCs after coculture with BMMSCs using MeRIP-seq. Compared to aged hGCs, hGCs cocultured with BMMSCs had 412 significantly upregulated genes and 405 notably downregulated genes (|log2FC|> 1, P value < 0.05) (Supplementary Fig. 3A), and the PPI networks are presented in Fig. 5A and B. A functional network analysis showed that the 412 genes were involved in regulation of mitotic sister chromatid segregation, positive regulation of protein localization to endosomes, and cellular senescence (Supplementary Fig. 3B). The KEGG pathway analysis mainly identified enrichment of the terms cytokine-cytokine receptor, pentose phosphate pathway, rheumatoid arthritis, and TNF signaling pathway (Fig. 5C). In addition, the 405 genes were involved in purine nucleoside triphosphate biosynthetic processes, neural crest cell migration, and embryonic camera-type eye formation (Supplementary Fig. 3C), and were significantly enriched in the terms nonhomologous end-joining, the Hippo signaling pathway, sphingolipid metabolism, and the homologous recombination signaling pathway (Fig. 5D). The results suggested that the main enriched signaling pathways of the 817 DEGs may play a defining role in the ability of BMMSCs to reverse hGC ageing.

## Correlation Analysis between Differential m6A Peaks and Differentially Expressed mRNAs

**Fig. 6:**
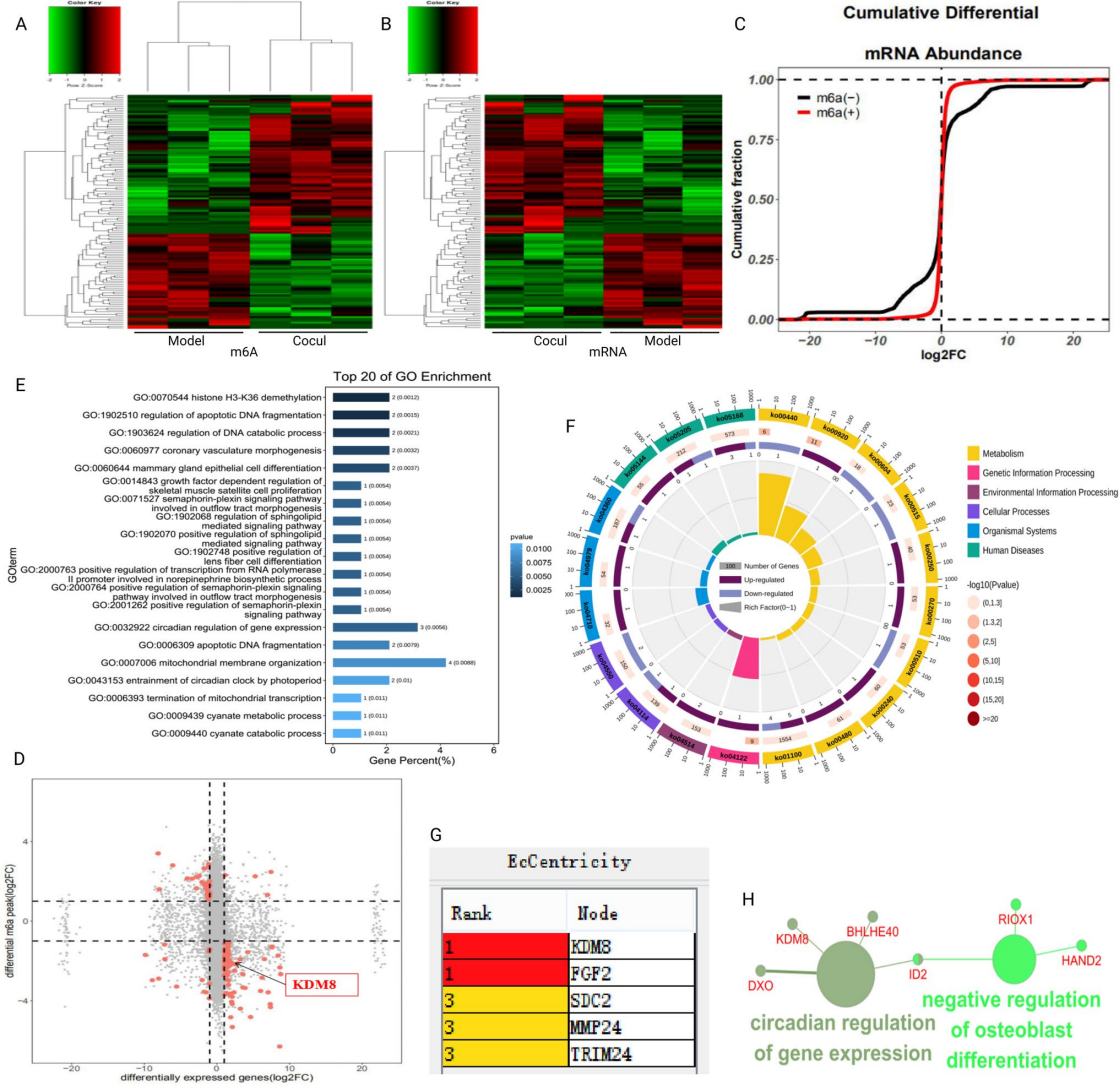
The correlation between differential m6A peaks and differentially expressed mRNAs. **A-B** Heat map of differential m6A peaks and differentially expressed mRNAs. **C** The cumulative differential mRNA abundance. **D** Four-quadrant diagram showing correlations between m6A peaks and mRNAs. **E** Top 20 enriched GO terms. **F** Top 20 enriched KEGG pathways. **G** PPI network showing the top 5 hub genes. **H** Pathway and functional enrichment analyses of KDM8

Correlation analyses of altered m6A peaks with differentially expressed mRNAs (|log2FC|> 1, P value < 0.05) were performed to identify the key genes through which BMMSCs affect the m6A methylation modification in hGCs ageing (Fig. 6A, B). The cumulative differential mRNA abundance is shown in Fig. 6C. We identified 42 hypermethylated m6A peaks in mRNAs that were significantly upregulated (3) or downregulated (39), while 88 hypomethylated m6A peaks in mRNAs were significantly upregulated (74) or downregulated (14) (Fig. 6D). Next, 130 genes that showed significant changes in both m6A modification and RNA expression levels were subjected to GO, pathway and PPI network analyses. The GO analyses of processes associated with the 130 gene sets are shown in detail in Fig. 6E and identified numerous linked functional processes and pathways. We found that the top 3 GO terms of histone H3-K36 demethylation (KDM8 and RIOX1), regulation of apoptotic DNA fragmentation, and regulation of DNA catabolic process were enriched in the GO maps of aged hGCs cocultured with BMMSCs (Fig. 6E). Interestingly, the correlation of histone H3-K36 demethylation was consistent with the results of the GO analysis of DEGs (Supplementary Fig. 3B). In addition, the top 20 KEGG pathways are shown in Fig. 6F and were significantly enriched in metabolism, genetic information processing, and environmental information processing. Moreover, the results of the EcCenticity analysis showed that KDM8 ranked first in the PPI network (Fig. 6G) and was involved in negative regulation of osteoblast differentiation and circadian regulation of gene expression (Fig. 6H). These observations indicate that KDM8 with the m6A modification may play important roles in reversing hGC ageing through interference by BMMSCs.

## The Changes of KDM8 in BMMSCs Reversing hGCs Aging and after Inhibited the Expression of FTO

**Fig. 7:**
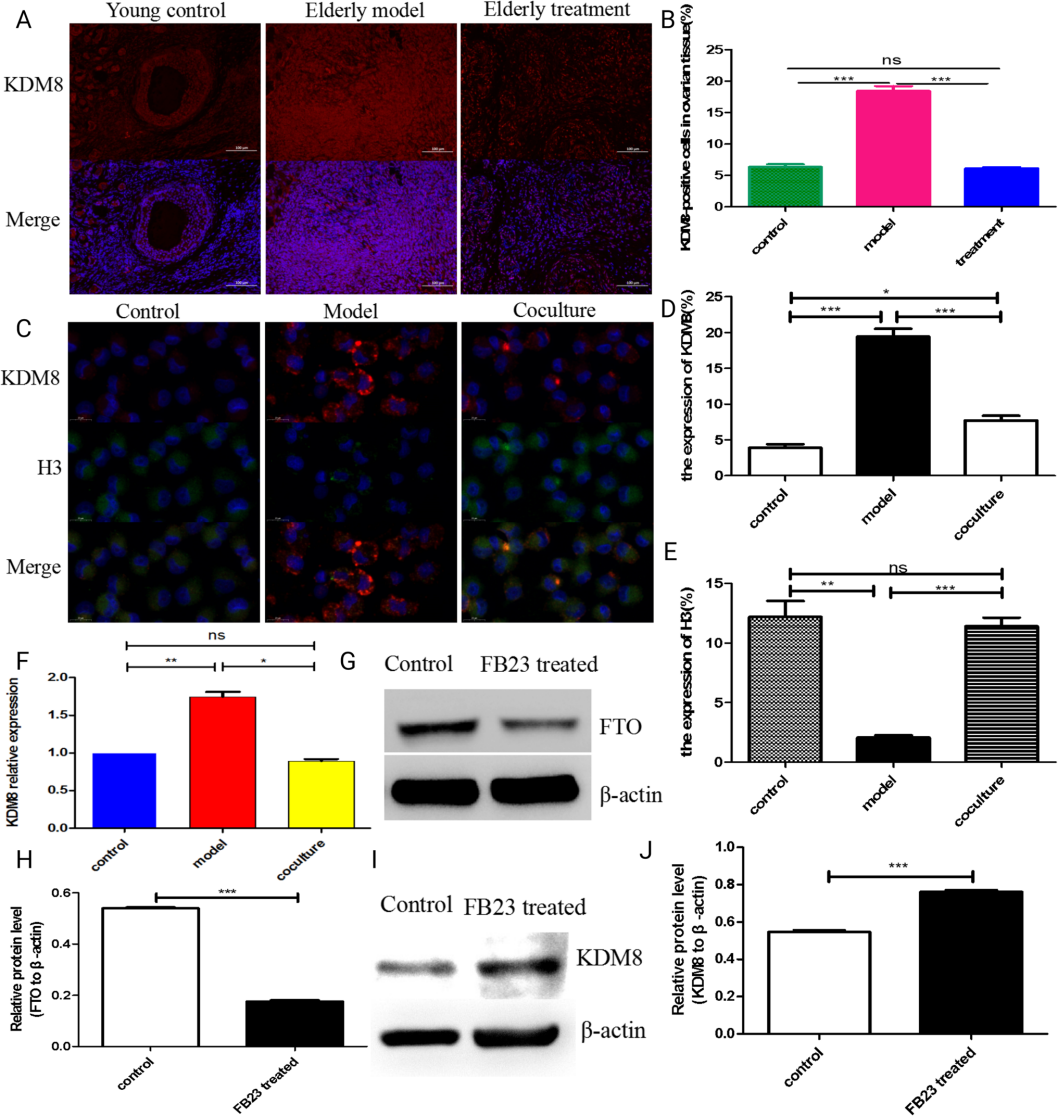
The changes in KDM8 after treatment with BMMSCs. **A-B** Immunofluorescence staining detected the expression of KDM8 in ovarian tissues of macaques. **C-E** Immunofluorescence costaining of KDM8 and H3. **F** q-PCR detected the mRNA expression of KDM8 in hGCs. **G-H** Western blot detected the protein expression of FTO. **I-J** Western blot detected the protein expression of KDM8 after inhibited FTO expression

KDM8 (lysine demethylase 8) is an epigenetic repressive mark and important cell cycle regulator that functions as a transcriptional activator by inhibiting HDAC recruitment via the demethylation of H3K36me2 [34], and is involved in osteoblast differentiation [35]. Methylation modifications of histones play critical roles in regulating gene expression, the cell cycle, genome stability, and nuclear structure; therefore, we explored the regularity of KDM8 in hGCs and ovarian tissue, and the intervening effect of BMMSCs. Compared with the aged model group, KDM8 protein expression was downregulated in the ovarian tissue of macaques after BMMSC treatment (Fig. 7A, B). According to a previous study, histone H3 is a major target of KDM8 cleavage activity, and the N-tail of H3 is proteolytically cleaved between K9 and S10 by KDM8 following DNA damage [36]. Subsequently, our immunofluorescence costaining of KDM8 and H3 results showed that the expression of the histone H3 protein was upregulated in aged hGCs after coculture with BMMSCs (Fig. 7C, D, E), which exhibited a change contrary to KDM8. In addition, both the KDM8 mRNA were downregulated in aged hGCs after coculture with BMMSCs (Fig. 7F). FB23 as an effective and selective inhibitor of FTO demethylase activity [37], therefore, FB23 was used to inhibit FTO and explore whether FTO downregulation influenced KDM8 expression, our results showed that FTO significantly downregulated after 30 μm FB23 treated 48 h (Fig. 7G, H), and KDM8 was upregulated after the selective inhibition of FTO in normal hGCs (Fig. 7I, J). These results imply that the regulatory mechanisms by which BMMSCs reverse hGC ageing involve the upregulation of FTO expression to reduce m6A modification of KDM8 to promote the protein expression of KDM8, and then KDM8 mediates changes in H3.

## Discussion

BMMSCs have multidirectional differentiation potential, a strong self-renewal ability, the ability to combat oxidative stress and inflammation, and secrete various cytokines, which have been postulated to play a key role in reversing ovarian ageing. Moreover, hGCs are the most important auxiliary cells and provide support and nutrition for follicles and oocytes [38]. Therefore, we used BMMSCs cocultured with aged hGCs in a Transwell system to explore the interaction mechanism. Interestingly, the structure and function of the ovary significantly improved after BMMSC treatment, the levels of β-galactosidase and ROS decreased, proliferation increased, and the expression of the P53 protein was downregulated after aged hGCs were cocultured with BMMSCs, indicated that BMMSCs restored the levels of the aforementioned factors related to ageing to normal levels comparable to those in the model group. Additionally, a model of ageing hGCs was successfully established, and BMMSCs reversed hGC ageing.

The m6A modification has been reported as a novel epigenetic modification that is strongly associated with ageing and various ageing-related diseases [39, 40]. However, researchers have not clearly determined whether BMMSCs reverse hGC ageing through the m6A modification. In our study, compared to the control group, the expression of FTO was downregulated and the overall m6A levels increased in the model group, consistent with a previous study showing that increased m6A levels in hGCs mediate faster aging-related phenotypes that result in ovarian ageing [22]. The expression of FTO was upregulated and the overall m6A levels decreased after BMMSC treatment. In addition, we identified 797 altered m6A peaks after BMMSC treatment, and they were significantly enriched in chromatin modification, regulation of transcription, DNA-templated, and cell cycle, which were associated with ovarian ageing. Additionally, in KEGG pathway analyses, the spliceosome, Epstein-Barr virus infection, and thyroid hormone signalling pathway were significantly correlated with genes that showed m6A peaks in aged hGCs, suggesting that m6A peaks play a key role in regulating ovarian ageing.

In the analysis of the mRNA transcriptional profile, we detected 817 genes with altered expression after aged hGCs were cocultured with BMMSCs, of which 412 genes were upregulated and 405 genes were downregulated. The 412 genes function in the regulation of mitotic sister chromatid segregation, positive regulation of protein localization to endosomes, and cellular senescence, and the 405 genes were significantly associated with embryonic placenta morphogenesis and fertilization, which have been linked to epigenetic modification and female fertilization function. These results suggested that BMMSCs may interact with aged hGCs by regulating the transcriptional profile of those mRNAs to reverse ovarian ageing.

By combining MeRIP-seq and RNA-seq data, we identified 42 hypermethylated m6A peaks in mRNAs that were significantly upregulated (3) or downregulated (39) and 88 hypomethylated m6A peaks in mRNAs that were significantly upregulated (74) or downregulated (14). We found that histone H3-K36 demethylation (KDM8 and RIOX1) ranked first in the GO analysis, and the enriched pathways mainly included metabolism, genetic information processing, and environmental information processing. Interestingly, the correlation results for histone H3-K36 demethylation were consistent with the GO analysis of the DEGs, and the signalling pathways of KDM8 were involved in negative regulation of osteoblast differentiation. This result is consistent with the finding that cellular senescence is mediated by the positive regulation of osteoblast differentiation, and with the research findings that m6A is important to maintain the bone mass and functions to protect osteoblasts from the ROS-mediated cell ageing process [41]. KDM8 is a epigenetic repressive mark and important cell cycle regulator, high expression of KDM8 suppresses migration and proliferation [42–44], consistent with our results that KDM8 was upregulated in the model group but was downregulated after BMMSC treatment, at the same time, the expression of H3 showed a change contrary to KDM8. Additionally, KDM8 subsequently dwonregulated after FTO was inhibited, indicated that the expression of KDM8 was significantly related to m6A methylation modification. Therefore, we speculate KDM8 may be a component of a novel regulatory mechanism in BMMSCs to reverse hGC ageing.

In summary, BMMSCs reverse changes in hGC ageing-related indexes, upregulate the expression of FTO, and decrease the overall m6A level. The m6A methylation modification plays an important role in the ability of BMMSCs to reverse hGC ageing, and the FTO regulated KDM8-mediates changes in H3 may represent a novel regulatory mechanism in BMMSCs to reverse hGC ageing.

## Conclusion

i. In the ageing model of hGCs, hGCs were treated with 273 mM H_2_O_2_ for 24 h, leading to increased levels of β-galactosidase and ROS, reduced proliferation, and increased expression of P53 to induce hGC ageing, while BMMSCs reversed the changes in the aforementioned factors related to ageing.
ii. BMMSCs significantly upregulated the expression of FTO and reduced overall levels of the m6A RNA methylation modification.
iii. The m6A methylation modification mainly occurred in the 3’UTR and CDS of mRNAs to regulate their expression and subsequently induce or suppress hGC ageing, and upregulation of FTO expression to reduce m6A modification of KDM8 to promote the protein expression of KDM8, and then KDM8 mediates changes in H3, which may represent a novel regulatory mechanism in BMMSCs to reverse hGC ageing.

## Materials and Methods

### Materials

BMMSCS were provided by the Basic Medical Laboratory of the 920th Hospital of Joint Logistics Support Force of PLA, The Transfer Medicine Key Laboratory of Cell Therapy Technology of Yunan Province, and The Integrated Engineering Laboratory of Cell Biological Medicine of State and Regions. hGCs were purchased from Bainer Chuanglian Biotechnology Co., Ltd.

### Induction of hGCs Ageing

When hGCs reached 80% confluence in 6-well plates, the model group was treated with 273 mM H_2_O_2_ for 24 h. β-Galactosidase staining was used to detect the expression of β-galactosidase [45], DHE staining was performed to detect the level of ROS [46], and BrdU staining were performed to observe proliferation [47]. Immunohistochemical staining was performed to detect the expression of the P53 protein [48].

### Aged hGCs Cocultured with BMMSCs

A total of 10^4^ hGCs were added to the lower chamber of the Transwell plate. When the confluence rate reached 80%, 273 mM H_2_O_2_ was incubated with the cells for 24 h. Then, the medium was changed, and 10^4^ BMMSCs at P4 were added to the upper chamber of the Transwell for the model and coculture groups, which were cocultured for 48 h, and β-galactosidase staining, DHE staining, BrdU staining, and immunohistochemical staining were performed to detect the relative ageing indexes.

### Colorimetry

The overall methylation level detection kit (EpiQuik™ m6A RNA Methylation Quantification Kit, Colorimetric, Epigentek) quantifies m6A ribonucleic acid methylation by extracting total RNA for m6A RNA capture and then measuring the signal at 450 nm using a microplate reader.

### MeRIP-seq

hGCs in the control, model, and coculture groups were collected, TRIzol was added to the lysate, and total RNA was extracted for reverse transcription. High-throughput sequencing was performed to obtain raw data, which were then extracted and quality controlled. Processing yielded clean reads, and FastQC was used to analyse the quality of sequencing data and obtain information. Mapping analyses identified the source of the sequencing sequence, its position in the genome, and unique mapped reads. HISAT2 software was used to compare the filtered clean reads with the reference genome of the corresponding species of the sample to obtain unique mapped reads for further analysis. The tdf file or bigwig file was converted from the processed bam file after standardization and used for IGV or Genome Browser (UCSC) visualization. ExomePeak was used to verify the quality of the data and the enrichment of short sequences in the genome. Peak annotation was analysed, and the gene structure and overall distribution characteristics of the peak were determined to draw metagene plots and pie diagrams. HOMER (http://homer.ucsd.edu/homer/ngs/peakMotifs.html) software was used to perform motif analysis of the peaks.

Differential peak analysis first identifies reads enriched in binding sites and then checks whether these sites have differential methylation modifications under the two experimental conditions and statistical tests. Differential m6A peak-modified genes were analysed with STRING and Cytoscape. The m6A-modified genes were analysed with GO and KEGG analyses, the logarithmic result of a significant P-value was used for visualization, and the first 20 terms were selected to draw a bubble chart.

### Analysis of the Correlation of m6A Modifications with RNA Expression

Genome version GRCh38_Ensembl91_year_2017 was used with htseq-count to count the number of reads in some units of the genome. Differential expression analysis was performed with DESeq2, and the significantly different gene criteria were as follows: 1. |log2FC|> 1; 2. P-value < 0.05. Cluster analysis of the DEGs was performed. A normalized expression table of the selected differential genes was used as the input file. Fisher’s test was used to calculate the significance level (P-value) of each GO term and pathway. The m6A-modified genes and the mRNA expression level changes were analysed for correlation, and some key genes related to the ageing phenotype were identified for follow-up research. A cumulative distribution diagram and a four-quadrant diagram were generated to show the correlation analysis results.

### Reverse Transcription and Quantitative PCR (RT-PCR)

Total RNA was extracted from hGCs using TRIzol reagent (Invitrogen, USA) and reverse transcribed into cDNAs using the High-Capacity RNA to cDNA Kit (Goldenstar^TM^RT6, China); the CFX96™ real-time system was used to perform Q-PCR with SYBR Green Master Mix (TsingKe Biotech Co., China). The primer sequences for KDM8 are F: 5,-ACAAAGAAAGCAAGGGCGGA-3, and R: 5,-ACCTCGAACCAACTTCCACT-3,, and the results were normalized to GAPDH levels.

### Western Blot

Cells from each group were collected, total protein was extracted, and the BCA protein concentration assay kit was used to measure the protein concentration. The protein samples were mixed with 5X reducing protein loading buffer at a ratio of 4:1, denatured in a boiling water bath for 15 min, and separated on SDS-PAGE gels. Proteins were transferred to a membrane at a constant current of 300 mA before the membrane was blocked with skim milk for 30 min. Next, the primary antibody (FTO or KDM8) was added and incubated at 4 °C overnight; the membrane was washed three times with TBST for 5 min each The secondary antibody was added and incubated for 30 min, and the membrane was washed three times with TBST for 5 min each before development and fixation. ImageJ software was used to analyse the grayscale values of the protein bands.

### Positive Cell Count and Statistical Analysis

The FTO-, P53-, BrdU-, ROS-, KDM8-, and H3-positive GCs were observed under an Olympus fluorescence microscope. Three visual fields were randomly selected from each group, and ImageJ software was used to count and analyse the number of positive cells per unit area. SPSS l8.0 software was used to analyse the experimental results, and the data are presented as means ± standard deviations. The differences between the two groups were analysed using an independent sample t-test, and the differences among multiple groups were analysed using single factor analysis of variance. P < 0.05 indicates that the difference was statistically significant.

## Supplementary Information

Below is the link to the electronic supplementary material.

Supplementary file1 (DOCX 825 KB)

## Data Availability

All data generated or analysed during this study are included in this published article.

## Abbreviations

BMMSCs: Bone marrow mesenchymal stem cells
hGC: Human granulosa cell
MeRIP-seq: M6A-modified RNA immunoprecipitation sequencing
KDM8: Lysine demethylase 8
m6A: N6-methyladenosine
DEGs: Differentially expressed genes

## References

[1] Barrera-Vazquez OS, Gomez-Verjan JC (2020). The unexplored world of human virome, mycobiome, and archaeome in aging. Journals of Gerontology. Series A, Biological Sciences and Medical Sciences.

[2] Grande G, Qiu C, Fratiglioni L (2020). Prevention of dementia in an ageing world: Evidence and biological rationale. Ageing Research Reviews.

[3] Khosla S, Farr JN, Tchkonia T, Kirkland JL (2020). The role of cellular senescence in ageing and endocrine disease. Nature Reviews. Endocrinology.

[4] Moolhuijsen, L., Visser, J. A. (2020). Anti-mullerian hormone and ovarian reserve: Update on assessing ovarian function. *The Journal of Clinical Endocrinology and Metabolism, 105*. 10.1210/clinem/dgaa513 PMC7486884 32770239

[5] Fan X, Bialecka M, Moustakas I, Lam E, Torrens-Juaneda V, Borggreven NV (2019). Single-cell reconstruction of follicular remodeling in the human adult ovary. Nature Communications.

[6] Xie, R., Liu, M. (2022). Relationship between non-alcoholic fatty liver disease and degree of hepatic steatosis and bone mineral density. *Front Endocrinol (Lausanne)13*:857110 10.3389/fendo.2022.857110 PMC8964007 35360054

[7] Li CJ, Lin LT, Tsai HW, Chern CU, Wen ZH, Wang PH (2021). The molecular regulation in the pathophysiology in ovarian aging. Aging & Disease.

[8] Helvaci N, Yildiz BO (2020). Cardiovascular health and menopause in aging women with polycystic ovary syndrome. Expert Review of Endocrinology and Metabolism.

[9] Tesarik, J., Galan-Lazaro, M., Mendoza-Tesarik, R. (2021). Ovarian aging: Molecular mechanisms and medical management. *International Journal of Molecular Sciences, 22*. 10.3390/ijms22031371 PMC7866420 33573050

[10] Yang L, Chen Y, Liu Y, Xing Y, Miao C, Zhao Y (2020). The role of oxidative stress and natural antioxidants in ovarian aging. Frontiers in Pharmacology.

[11] Busnelli, A., Navarra, A., Levi-Setti, P. E. (2021). Qualitative and quantitative ovarian and peripheral blood mitochondrial DNA (mtDNA) alterations: Mechanisms and implications for female fertility. *Antioxidants (Basel), 10*. 10.3390/antiox10010055 PMC7824846 33466415

[12] Arthur, A., Gronthos, S. (2020). Clinical application of bone marrow mesenchymal stem/stromal cells to repair skeletal tissue. *International Journal of Molecular Sciences, 21*. 10.3390/ijms21249759 PMC7767389 33371306

[13] Hernigou P, Delambre J, Quiennec S, Poignard A (2021). Human bone marrow mesenchymal stem cell injection in subchondral lesions of knee osteoarthritis: A prospective randomized study versus contralateral arthroplasty at a mean fifteen year follow-up. International Orthopaedics.

[14] Kim KH, Kim EY, Kim GJ, Ko JJ, Cha KY, Koong MK (2020). Human placenta-derived mesenchymal stem cells stimulate ovarian function via miR-145 and bone morphogenetic protein signaling in aged rats. Stem Cell Research & Therapy.

[15] Fabregues F, Ferreri J, Mendez M, Calafell JM, Otero J, Farre R (2020). In vitro follicular activation and stem cell therapy as a novel treatment strategies in diminished ovarian reserve and primary ovarian insufficiency. Frontiers in Endocrinology (Lausanne).

[16] El-Derany MO, Said RS, El-Demerdash E (2021). Bone marrow-derived mesenchymal stem cells reverse radiotherapy-induced premature ovarian failure: Emphasis on signal integration of TGF-beta, Wnt/beta-Catenin and hippo pathways. Stem Cell Reviews and Reports.

[17] Buigues A, Marchante M, de Miguel-Gomez L, Martinez J, Cervello I, Pellicer A (2021). Stem cell-secreted factor therapy regenerates the ovarian niche and rescues follicles. American Journal of Obstetrics and Gynecology.

[18] Wang Z, Yang T, Liu S, Chen Y (2020). Effects of bone marrow mesenchymal stem cells on ovarian and testicular function in aging Sprague-Dawley rats induced by D-galactose. Cell Cycle.

[19] Karthiya R, Khandelia P (2020). m6A RNA methylation: Ramifications for gene expression and human health. Molecular Biotechnology.

[20] Li, Y., Gu, J., Xu, F., Zhu, Q., Chen, Y., Ge, D., et al. (2021). Molecular characterization, biological function, tumor microenvironment association and clinical significance of m6A regulators in lung adenocarcinoma. *Briefings in Bioinformatics, 22*. 10.1093/bib/bbaa225 33003204

[21] Mapperley, C., van de Lagemaat, L. N., Lawson, H., Tavosanis, A., Paris, J., Campos, J., et al. (2021). The mRNA m6A reader YTHDF2 suppresses proinflammatory pathways and sustains hematopoietic stem cell function. *Journal of Experimental Medicine, 218*. 10.1084/jem.20200829 PMC7653684 33156926

[22] Jiang ZX, Wang YN, Li ZY, Dai ZH, He Y, Chu K (2021). The m6A mRNA demethylase FTO in granulosa cells retards FOS-dependent ovarian aging. Cell Death & Disease.

[23] Zhang S, Deng W, Liu Q, Wang P, Yang W, Ni W (2020). Altered m(6) A modification is involved in up-regulated expression of FOXO3 in luteinized granulosa cells of non-obese polycystic ovary syndrome patients. Journal of Cellular and Molecular Medicine.

[24] Zhang J, Ao Y, Zhang Z, Mo Y, Peng L, Jiang Y (2020). Lamin A safeguards the m(6) A methylase METTL14 nuclear speckle reservoir to prevent cellular senescence. Aging Cell.

[25] Trauner M, Gindin Y, Jiang Z, Chung C, Subramanian GM, Myers RP (2020). Methylation signatures in peripheral blood are associated with marked age acceleration and disease progression in patients with primary sclerosing cholangitis. JHEP Reports.

[26] Yin Z, Park R, Choi BM (2020). Isoparvifuran isolated from Dalbergia odorifera attenuates H2O2-induced senescence of BJ cells through SIRT1 activation and AKT/mTOR pathway inhibition. Biochemical and Biophysical Research Communications.

[27] Jiang F, Xu XR, Li WM, Xia K, Wang LF, Yang XC (2020). Monotropein alleviates H2O2induced inflammation, oxidative stress and apoptosis via NFkappaB/AP1 signaling. Molecular Medicine Reports.

[28] Quan Y, Xin Y, Tian G, Zhou J, Liu X (2020). Mitochondrial ROS-modulated mtDNA: A potential target for cardiac aging. Oxidative Medicine and Cellular Longevity.

[29] Madreiter-Sokolowski CT, Thomas C, Ristow M (2020). Interrelation between ROS and Ca(2+) in aging and age-related diseases. Redox Biology.

[30] Jiang ZX, Wang YN, Li ZY, Dai ZH, He Y, Chu K (2021). Correction: The m6A mRNA demethylase FTO in granulosa cells retards FOS-dependent ovarian aging. Cell Death & Disease.

[31] Qin L, Min S, Shu L, Pan H, Zhong J, Guo J (2020). Genetic analysis of N6-methyladenosine modification genes in Parkinson's disease. Neurobiology of Aging.

[32] Sendinc E, Valle-Garcia D, Dhall A, Chen H, Henriques T, Navarrete-Perea J (2019). PCIF1 catalyzes m6Am mRNA methylation to regulate gene expression. Molecular Cell.

[33] Liu J, Dou X, Chen C, Chen C, Liu C, Xu MM (2020). N (6)-methyladenosine of chromosome-associated regulatory RNA regulates chromatin state and transcription. Science.

[34] Wilkins SE, Islam MS, Gannon JM, Markolovic S, Hopkinson RJ, Ge W (2018). JMJD5 is a human arginyl C-3 hydroxylase. Nature Communications.

[35] Munehira Y, Yang Z, Gozani O (2017). Systematic analysis of known and candidate lysine demethylases in the regulation of myoblast differentiation. Journal of Molecular Biology.

[36] Shen J, Xiang X, Chen L, Wang H, Wu L, Sun Y (2017). JMJD5 cleaves monomethylated histone H3 N-tail under DNA damaging stress. EMBO Reports.

[37] Huang Y, Su R, Sheng Y, Dong L, Dong Z, Xu H (2019). Small-Molecule Targeting of Oncogenic FTO Demethylase in Acute Myeloid Leukemia. Cancer Cell.

[38] Ding Y, Zhu Q, He Y, Lu Y, Wang Y, Qi J (2021). Induction of autophagy by Beclin-1 in granulosa cells contributes to follicular progesterone elevation in ovarian endometriosis. Translational Research.

[39] Shafik AM, Zhang F, Guo Z, Dai Q, Pajdzik K, Li Y (2021). N6-methyladenosine dynamics in neurodevelopment and aging, and its potential role in Alzheimer's disease. Genome Biology.

[40] Chen X, Wang J, Tahir M, Zhang F, Ran Y, Liu Z (2021). Current insights into the implications of m6A RNA methylation and autophagy interaction in human diseases. Cell & Bioscience.

[41] Zhang Q, Riddle RC, Yang Q, Rosen CR, Guttridge DC, Dirckx N (2019). The RNA demethylase FTO is required for maintenance of bone mass and functions to protect osteoblasts from genotoxic damage. Proceedings of the National Academy of Sciences.

[42] Yao Y, Zhou WY, He RX (2019). Down-regulation of JMJD5 suppresses metastasis and induces apoptosis in oral squamous cell carcinoma by regulating p53/NF-kappaB pathway. Biomedicine & Pharmacotherapy.

[43] Fuhrmann D, Mernberger M, Nist A, Stiewe T, Elsasser HP (2018). Miz1 Controls Schwann Cell Proliferation via H3K36(me2) Demethylase Kdm8 to Prevent Peripheral Nerve Demyelination. Journal of Neuroscience.

[44] Fuhrmann D, Elsasser HP (2018). Schwann cell Myc-interacting zinc-finger protein 1 without pox virus and zinc finger: Epigenetic implications in a peripheral neuropathy. Neural Regeneration Research.

[45] Truman AM, Tilly JL, Woods DC (2017). Ovarian regeneration: The potential for stem cell contribution in the postnatal ovary to sustained endocrine function. Molecular and Cellular Endocrinology.

[46] Li W, Li Y, Zhao Y, Ren L (2020). The protective effects of aloperine against ox-LDL-induced endothelial dysfunction and inflammation in HUVECs. Artificial Cells, Nanomedicine, and Biotechnology.

[47] Ding C, Zou Q, Wang F, Wu H, Chen R, Lv J (2018). Human amniotic mesenchymal stem cells improve ovarian function in natural aging through secreting hepatocyte growth factor and epidermal growth factor. Stem Cell Research & Therapy.

[48] Liu YA, Ji JX, Almadani N, Crawford RI, Gilks CB, Kinloch M (2021). Comparison of p53 immunohistochemical staining in differentiated vulvar intraepithelial neoplasia (dVIN) with that in inflammatory dermatoses and benign squamous lesions in the vulva. Histopathology.

