# Supplementary material for: Bone Marrow Mesenchymal Stem Cells Reversed Ovarian Aging-related m6A RNA Methylation Modification Profile in Aged Granulosa Cells
Source: Stem Cell Rev Rep. 2023 Jan 7;19(4):953–67. doi: 10.1007/s12015-022-10485-y (PMC10185602; doi:10.1007/s12015-022-10485-y)
Supplement: Supplementary file 1 — Supplementary file1 (DOCX 825 KB) [file 12015_2022_10485_MOESM1_ESM.docx]

**Supplementary**


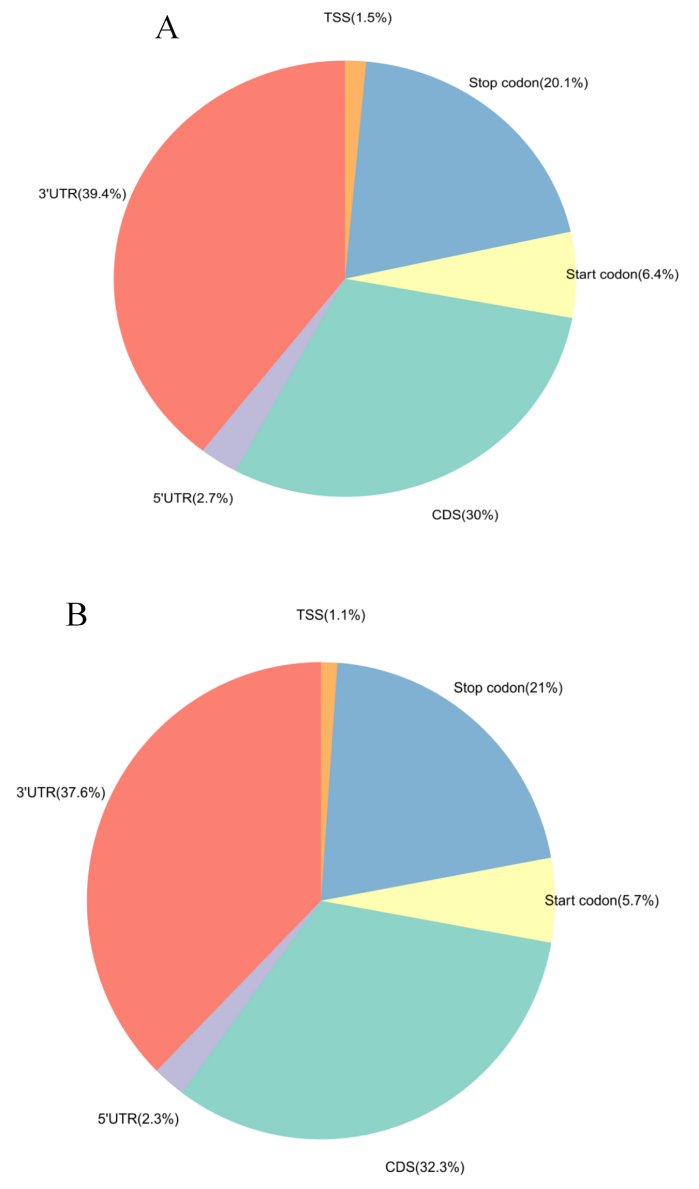


**Figure 1.** **A-B** Pie charts showing the distribution of m6A peaks in the model and coculture groups.


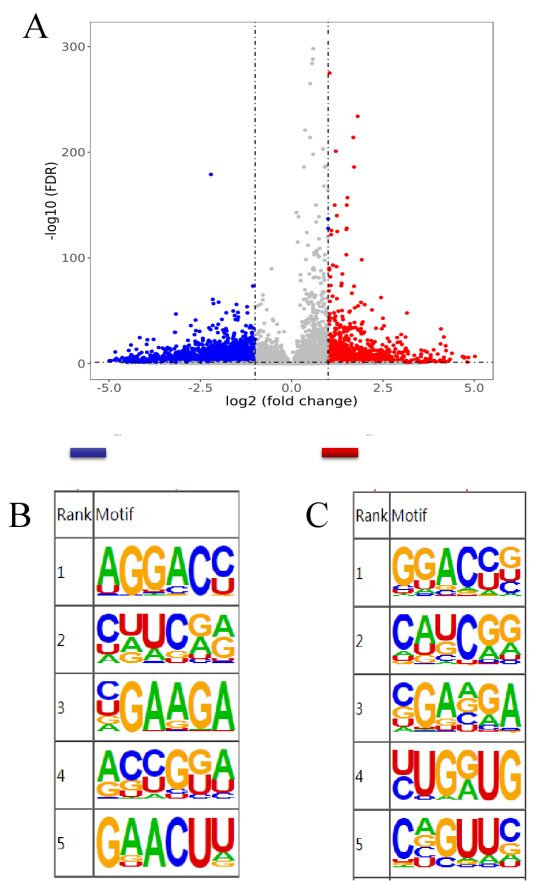


**Figure 2. A** Volcano plot showing the differential m6A peaks in the model and coculture groups. **B** the classic motif of model group. **C** the classic motif of cocolture group.

**
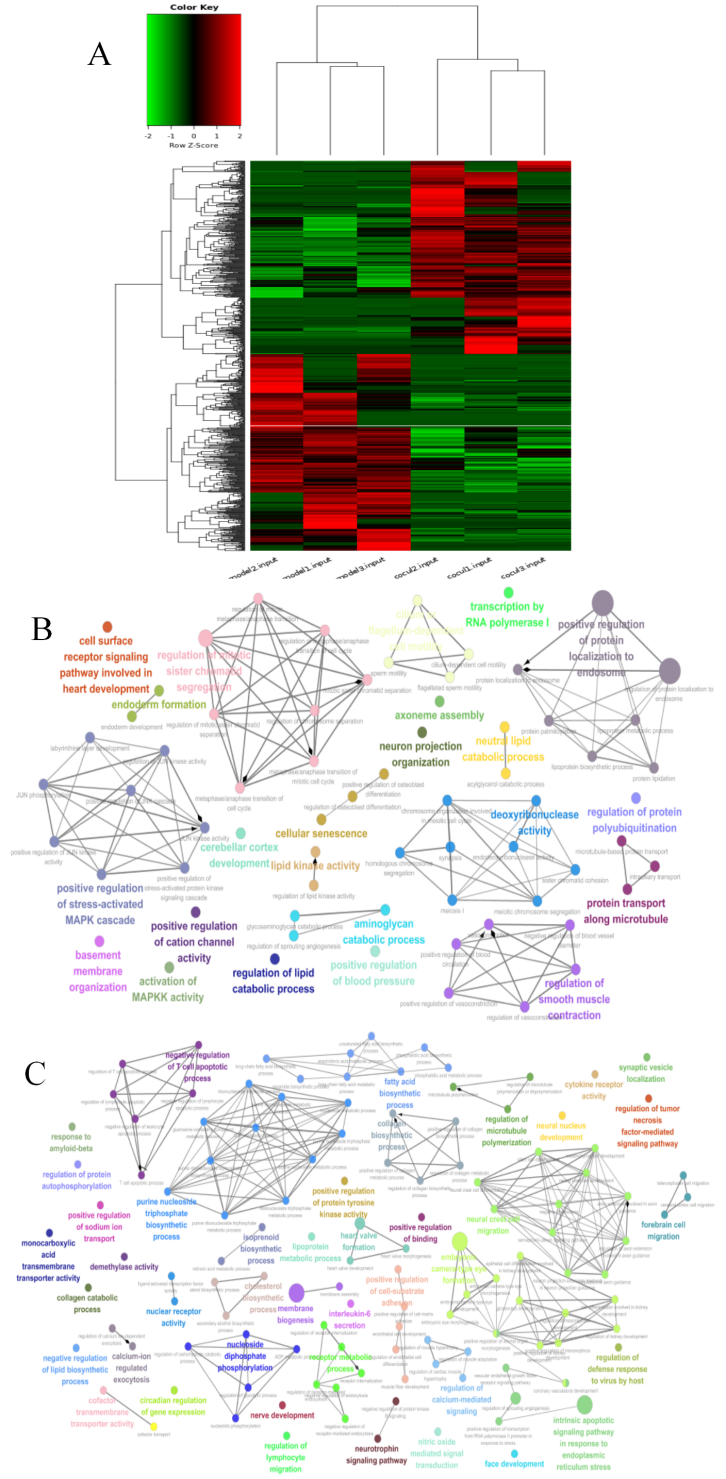
**

**Figure 2. A** Heat map showing the differential expression of RNAs in the model and coculture groups. **B** Functional interaction network of downregulated genes after cocultured with BMMSCs. **C** Functional interaction network ofdownregulated genes after cocultured with BMMSCs.
